# Utility of hybrid whole genome sequencing in assessing potential nosocomial VIM transmission

**DOI:** 10.1017/ash.2024.347

**Published:** 2024-08-05

**Authors:** David Burke James Mahoney, Xena Li, Patryk Aftanas, Christie Vermeiren, Robert Kozak, Kevin Katz, Finlay Maguire

**Affiliations:** 1 Department of Community Health and Epidemiology, Faculty of Medicine, Dalhousie University, Halifax, NS, Canada; 2 Faculty of Computer Science, Dalhousie University, Halifax, NS, Canada; 3 Shared Hospital Laboratory, Toronto, ON, Canada; 4 Sunnybrook Health Sciences Centre, Toronto, ON, Canada; 5 Infection Prevention and Control, North York General Hospital, Toronto, ON, Canada; 6 Department of Laboratory Medicine and Pathology, University of Toronto, Toronto, ON, Canada

## Abstract

Hybrid whole genome sequencing was used to investigate if nosocomial Verona integron-encoded metallo-β-lactamase (VIM) carbapenemase transmission occurred between two patients without epidemiological links or common pathogens. Challenges in genomic methodology and appropriate analytical depth for mobile carbapenemase outbreaks are described including how inappropriate choices can mislead results and impact infection control practices.

## Introduction

Verona integron-encoded metallo-β-lactamase (VIM) is a carbapenemase found worldwide but with increased concentration in Greece, Italy, and Spain.^1^ VIM is typically associated with *Pseudomonas spp.* (representing 53.2% of 4,731 VIM-positive isolates in the National Center for Biotechnology Information Pathogen Detection Project); however, it is also present in Enterobacterales such as *Klebsiella spp.* (18.9% of VIM-positive isolates)*, Enterobacter spp.* (17.5%), and *Escherichia coli* (3.9%).^2,3^

VIM is challenging for public health surveillance and infection prevention and control (IPAC) due to frequent lateral gene transfers (LGT) through a nested association with multiple mobile genetic elements (MGEs).^1^ Specifically, VIM genes are associated with a class 1 integron which is often also located on a plasmid.^1^ This compounds established issues with analyzing MGEs using short-read whole genome sequencing (WGS).^4^ Therefore, long-read WGS is required to accurately identify^5^ and track transmission of MGE-associated antimicrobial resistance (AMR) determinants.^6^ Unfortunately, despite increasing availability of WGS to clinical labs, there is a lack of standardization in analyses performed or their downstream reporting to IPAC teams.^7^

We demonstrate the utility of long-read WGS data to arrive at meaningful conclusions in a nosocomial LGT investigation and the challenges in implementing a standardized set of analyses to investigate the transmission of highly mobile AMR genes.

## Materials and methods

### Carbapenemase-producing enterobacterales (CPE) surveillance

Routine CPE surveillance by rectal swabs was performed in accordance with institutional policy for patients admitted to high-risk units (medicine, oncology and geriatric mental health), known history of antimicrobial-resistant organisms or recent admission to any hospital within the last 12 months. Clinical isolates undergo routine antimicrobial susceptibility testing and are screened for CPE by demonstrating non-susceptibility to ertapenem or meropenem. Suspected related isolates will then undergo WGS.

### DNA extraction and WGS

DNA extraction was performed using both easyMAG (bioMerieux, France) and plasmid mini-preps (QIAgen, Canada). Libraries were constructed using the Nextera DNA Prep Kit (Illumina, San Diego, USA) and sequenced with a MiniSeq High Output Reagent Kit (Illumina, San Diego, USA) with 149 bp paired-end reads, resulting in a mean 400–600x plasmid coverage depth.

A second set of libraries was constructed using the Rapid Barcoding Kit 96 v10 (Oxford Nanopore Technologies, UK) and sequenced on a GridION (Oxford Nanopore Technologies, UK) with high-accuracy basecalling, resulting in a mean 50–150x plasmid coverage depth.

Raw data was then deposited in the Sequence Read Archive for each sample under BioProject PRJNA1030769.

### Whole genome sequence data analysis

WGS long and short reads were quality-filtered and hybrid-assembled followed by AMR gene and MGE identification and comparative analysis. Full details of analysis, scripts, accession information, and results can be found in https://github.com/maguire-lab/vim_linkage_analysis (10.5281/zenodo.10032195).

## Results

### Infection prevention and control investigation

IPAC was alerted to two patients with VIM β-lactamase detected within the same week at an acute care 400-bed community hospital.

Routine clinical workup identified a CPE: VIM-bearing *Proteus mirabilis* in Patient A’s urine culture. Patient A was admitted to a medicine ward immediately following prolonged hospitalization in Greece, and the VIM was non-locally acquired as the urine was collected on admission. Patient A did not undergo any operations or invasive procedures including cystoscopy and colonoscopies.

Admission CPE surveillance identified a CPE VIM-bearing *Enterobacter soli* from Patient B’s rectal swab. A carbapenemase-producing non-Enterobacterales (CPnE) VIM-bearing *Pseudomonas putida* was also isolated from Patient B’s swab as it grew on the carbapenemase screening agar. Typically, CPnE would not be pursued in routine CPE surveillance; however, the *Pseudomonas putida* isolate was investigated due to concerns it contained the same VIM as the *Enterobacter soli*. Patient B had no foreign travel and was not known to be carbapenem resistant Enterobacterales colonized. Patient B tested positive on admission to a different medicine ward approximately 10 days following Patient A; however, Patient B had multiple admissions in the months prior including a 1 week overlap with Patient A’s stay, although on different units. Patient B also did not undergo any operations or invasive procedures during any admissions. A full investigation did not identify any epidemiologic link between the two patients including shared units, consecutive diagnostic imaging visits, common staff, or any invasive procedures.

Given VIM’s local rarity, there was concern of nosocomial transmission from Patient A to Patient B from the overlap in hospitalizations. That year, the hospital performed 10,000 CPE screens with 0.15% positivity rate; however, no other VIM were identified aside from Patient A and B. Both patients were independently managed; however, no outbreak was declared, while WGS was pending as no epidemiologic link was found. Unit prevalence testing and environmental sampling were not performed, and no further VIMs were identified from clinical and surveillance samples in the subsequent 12 months.

### Whole genome sequence analyses

**Figure 1.**
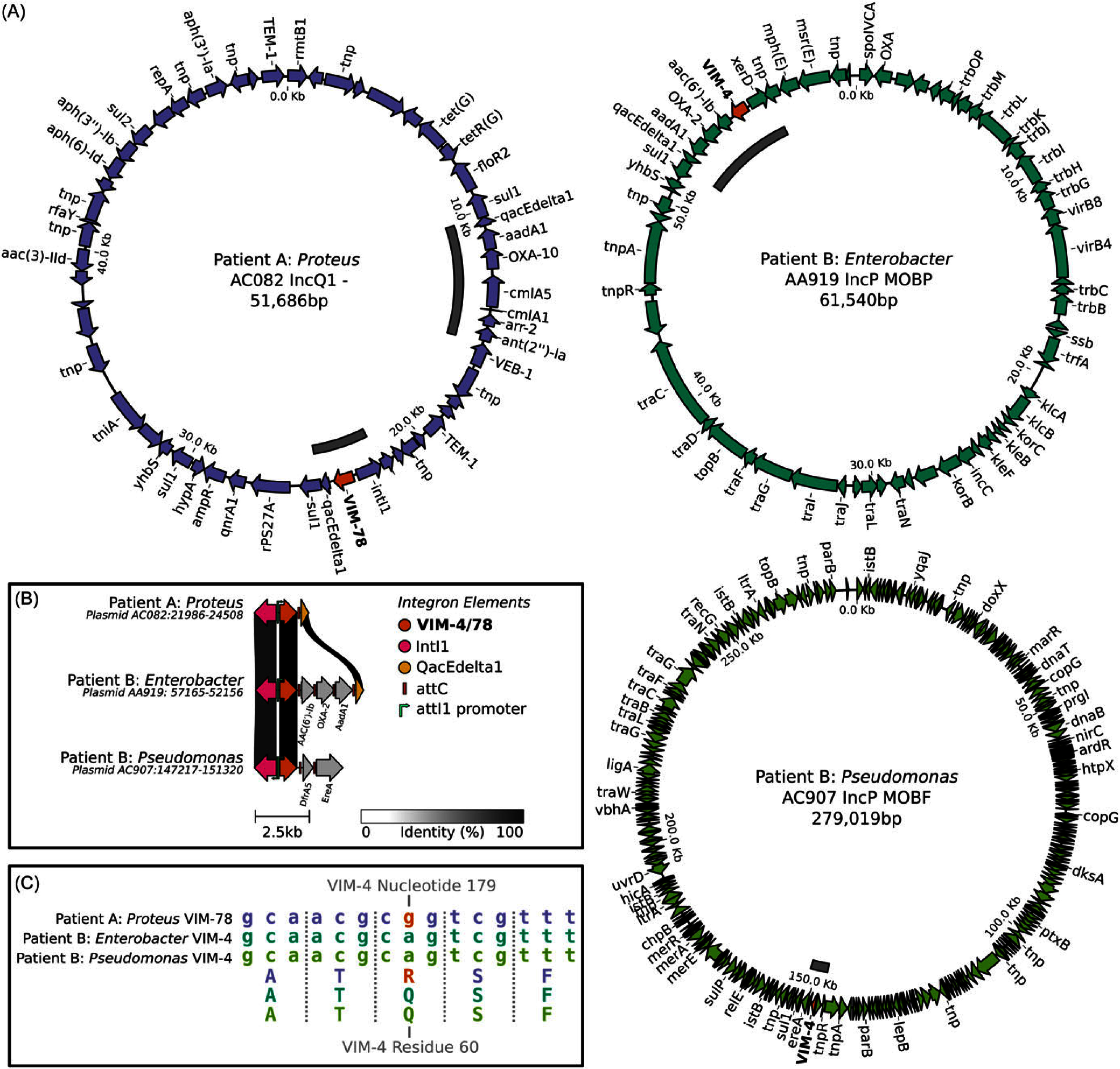
Overview of mobile VIM genomic analysis results. **A** The annotated VIM-bearing plasmids assembled from each of the isolates. The VIM gene is highlighted in orange and identified integrons in the plasmids marked using a dark gray bar in the inner circle. **B** The alignment and comparison of the three VIM-bearing integrons. The vertical ribbons indicate genes with shared sequence identity between the integrons (as per the % identity scale bar). **C** A comparison of the VIM alleles in each taxa in the form of a truncated alignment showing all the sequence differences.

Analysis of the VIM gene from each bacteria revealed identical VIM-4 genes in Patient B’s *Enterobacter soli* and *Pseudomonas putida*, while Patient A’s *Proteus mirabilis* had a VIM-78 gene differing by only one nucleotide from VIM-4 (Figure 1C). Plasmid analyses using long-read sequencing data indicated that Patient A’s *P. mirabilis* VIM-78 was located on a 51.6kb AC082 IncQ1 plasmid (Figure 1A). Patient B’s *E. soli* VIM-4 was located on a 61.5-kb AA919 IncP MOBP plasmid, and Patient B’s *P. putida* VIM-4 was located on a 279-kb AC907 IncP MOBF plasmid. Patient B’s *E. soli* VIM-4 was associated with a similar integron (shared QacEdelta1 gene and attC pattern) to Patient A’s *P. mirabilis* VIM-78, but differences in structure and gene complement do not support a recent LGT event (Figure 1B).

The two cases were ultimately suspected to be unrelated given two different VIM antimicrobial susceptibility testing phenotypes, lack of a clear epidemiological link between both patients, and differences in the associated MGEs.

## Discussion

Genomic analyses revealed that Patient A and Patient B harbored different VIM genes, but by just one nucleotide and therefore the possibility of nosocomial LGT remained (Figure 1C). Although all three VIM genes were found on plasmids, these plasmids were substantially different indicating no plasmid-based LGT had occurred (Figure 1A). However, the VIM genes were also associated with integron MGEs. Analysis revealed that although these integrons had similarities suggestive of some shared ancestry, they were distinct enough to rule out recent LGT (Figure 1B). Together, these analyses suggest LGT of the VIM gene was unlikely to have occurred between the organisms isolated from Patient A and Patient B.

**Figure 2.**
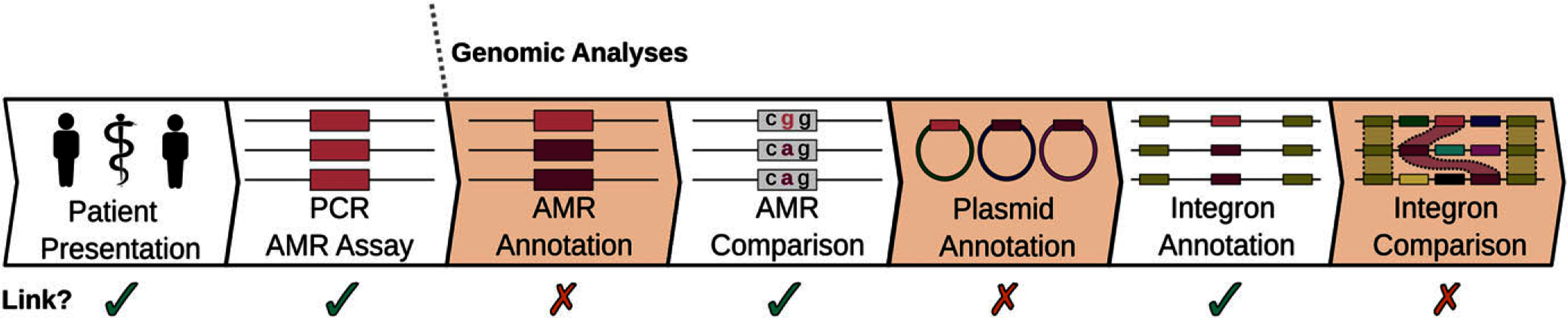
An overview of analyses and conflicting results during the investigation of a possible nosocomial transmission of a mobile VIM carbapenemase. Ticks and crosses (and associated box highlighting), respectively, represent information that supported or refuted potential linkage between patients.

If the genomic investigations were stopped earlier in the analyses (e.g. polymerase chain reaction, plasmid identification, and integron presence) conclusions could have been drawn that suggest LGT did occur or lack confidence to refute transmission (Figure 2). This could have led to an outbreak being declared with considerable use of healthcare resources and negative impact on patients. This study highlights that genomic complexity drives analytical complexity and that long-read WGS has high utility when reported to clinical stakeholders to inform decision-making. Ultimately, Patient A’s VIM was acquired out of country, but Patient B’s VIM could not be attributed to a source with known limitations in the lack of data in potential community transmission events and lack of surveillance in carbapenemase-producing non-Enterobacterales. Considering these VIM genes were likely not laterally transferred, continued CPE surveillance will be critical in the management of local VIM-producing organisms.
